# Evolutionary patterns of archaea predominant in acidic environment

**DOI:** 10.1186/s40793-023-00518-5

**Published:** 2023-07-18

**Authors:** Rafael Bargiela, Aleksei A. Korzhenkov, Owen A. McIntosh, Stepan V. Toshchakov, Mikhail M. Yakimov, Peter N. Golyshin, Olga V. Golyshina

**Affiliations:** 1 School of Natural Sciences and Centre for Environmental Biotechnology, Bangor University, Bangor, UK; 2 Kurchatov Center for Genome Research, NRC Kurchatov Institute, Moscow, Russia; 3 Institute of Polar Sciences, CNR, Messina, Italy

**Keywords:** Acid mine drainage (AMD), Mine-impacted environments, Parys Mountain, Acidophilic archaea, *Thermoplasmatales*, Microbial dark matter

## Abstract

**Background:**

Archaea of the order *Thermoplasmatales* are widely distributed in natural acidic areas and are amongst the most acidophilic prokaryotic organisms known so far. These organisms are difficult to culture, with currently only six genera validly published since the discovery of *Thermoplasma acidophilum* in 1970. Moreover, known great diversity of uncultured *Thermoplasmatales* represents microbial dark matter and underlines the necessity of efforts in cultivation and study of these archaea. Organisms from the order *Thermoplasmatales* affiliated with the so-called “alphabet-plasmas”, and collectively dubbed “E-plasma”, were the focus of this study. These archaea were found predominantly in the hyperacidic site PM4 of Parys Mountain, Wales, UK, making up to 58% of total metagenomic reads. However, these archaea escaped all cultivation attempts.

**Results:**

Their genome-based metabolism revealed its peptidolytic potential, in line with the physiology of the previously studied *Thermoplasmatales* isolates. Analyses of the genome and evolutionary history reconstruction have shown both the gain and loss of genes, that may have contributed to the success of the “E-plasma” in hyperacidic environment compared to their community neighbours. Notable genes among them are involved in the following molecular processes: signal transduction, stress response and glyoxylate shunt, as well as multiple copies of genes associated with various cellular functions; from energy production and conversion, replication, recombination, and repair, to cell wall/membrane/envelope biogenesis and archaella production. History events reconstruction shows that these genes, acquired by putative common ancestors, may determine the evolutionary and functional divergences of “E-plasma”, which is much more developed than other representatives of the order *Thermoplasmatales*. In addition, the ancestral hereditary reconstruction strongly indicates the placement of *Thermogymnomonas acidicola* close to the root of the *Thermoplasmatales*.

**Conclusions:**

This study has analysed the metagenome-assembled genome of “E-plasma”, which denotes the basis of their predominance in Parys Mountain environmental microbiome, their global ubiquity, and points into the right direction of further cultivation attempts. The results suggest distinct evolutionary trajectories of organisms comprising the order *Thermoplasmatales*, which is important for the understanding of their evolution and lifestyle.

**Supplementary Information:**

The online version contains supplementary material available at 10.1186/s40793-023-00518-5.

## Background

Low-pH environments hosting extreme acidophiles are ubiquitous on our planet [1]. Understanding the microbial diversity of these systems is important from positions of perception of life at its limits, estimation of metabolic potential, elements cycling and potential of biotechnological applications. Another aspect to be considered with acidic natural sites and microorganisms inhabiting them is the constant generation of intensive pollution: low pH metal-rich waters known as acid mine drainage (AMD) are a major environmental problem that is exacerbated by metal and coal mining. Currently known inhabitants of AMD include representatives of many different taxonomic groups across prokaryotes and eukaryotes [1]. Among archaea, the most prominent group of organisms populating AMD systems belong to the order *Thermoplasmatales* [*Euryarchaeota* or *Candidatus* Thermoplasmatota according to the Genome Taxonomy Database (GTDB)]. This taxonomic group includes a limited number of isolated and described members with a significant proportion of uncultured organisms, as revealed by metagenomic studies [1–3].

A recent shotgun metagenomic study of the site PM4, an acidic (pH 1.7) stream of Parys Mountain (Wales, UK) has revealed that one particular taxon within the order *Thermoplasmatales* accounted for 58% of total metagenomic reads [4]. These archaea designated as “E-plasma” have no cultured representatives and no taxonomic status, and were firstly detected in Iron Mountain, USA AMD site by [2]. Other *Thermoplasmatales* archaea inhabiting the Parys Mt site PM4 were identified as B_DKE phylotype (“*Scheffleriplasma harziensis*”), *Ferroplasma* spp. and *Cuniculiplasma* spp. Among uncultured groups within *Thermoplasmata*, *“*A-plasma” phylotype was also identified. Of note, however, above organisms were not detected in numbers as high as those of “E-plasma”, pointing at the predominance of this distinct group over other extreme acidophiles in the studied Parys Mt site [4]. The significant numbers of “E-plasma” reads suggest that the contribution of this archaeal phylotype to the turnover of carbon and other elements must be substantial. The distribution of sequences across the site showed predominance of this group on the surface of acidic stream associated with sediment, while the presence of “E-plasma” alongside archaeal numbers in the lotic fraction was insignificant [4]. Furthermore, “E-plasma” representatives were shown to be present across the depth of sediment of acidic stream (up to 25 cm) on Parys Mt site in numbers varying from 0.5 to 15% of prokaryotic SSU rRNA gene amplicon reads [5]. Besides, archaea of this phylotype were shown to inhabit other sites across Parys Mt [6].

Apart from Iron Mountain AMD site, similar archaea were also present across various acidic niches on Rio Tinto, Spain and acidic cave system Frasassi, Italy [2, 7, 8]. Other AMD and low pH geothermal sites with presence of similar phylotypes were identified in Fankou AMD outflow, and Tengchong geothermal area (China), Los Rueldos (Spain) and Los Azufres National Park (Mexico) [9, 10]. It should be added that Los Azufres geothermal area characterised by temperature 73.4 °C and pH 3.8. Furthermore, “E-plasma” has been found in less acidic systems, such as wetland sediments affected by coal deposits in the USA [11]. These records point at the ability of “E-plasma” to adapt to the broader pH and temperature ranges and therefore highlight the potential wider geographical ubiquity of these archaea.

Analysis of metagenome-assembled genomes (MAGs) reconstructed from metagenomic assemblies of Iron Mt site (USA) predicted “E-plasma”-related phylotypes to be heterotrophic and facultatively anaerobic, in concordance with general physiological features of the order *Thermoplasmatales* [12]. It should also be added that this group was a minor community constituent in Iron Mt, USA; Tengong geothermal area and Fankou mine, both China; Los Rueldos, Spain and Los Azufres geothermal area, Mexico [10, 12]. Notwithstanding and over again, these data contrast with the microbial community structure observed at Parys Mt, UK, where “E-plasma” was revealed as a numerically dominating phylotype among archaea and bacteria [4].

To further understand the backgrounds underlying the environmental success of this group of archaea, we conducted the analysis of the “E-plasma” genome assembled from metagenomic reads. We also performed a comparative genomic study of organisms present in the same environmental samples, of Parys Mt PM4 site that were representing major and minor groups, “E-plasma” and *Cuniculiplasma divulgatum*, correspondingly. Moreover, analysis of genomes of “E-plasma” variants from the two geographically distant AMD sites, Parys Mt, UK and Iron Mt, USA, and of other fully sequenced *Thermoplasmatales* members allowed the reconstruction of their evolution from phylogenomic ancestors.

## Materials and methods

### DNA extraction and sequencing

The environmental DNA extraction and sequencing were described previously [4].

### “E-plasma” binning workflow

Quality control of sequencing reads was performed with FastQC (https://www.bioinformatics.babraham.ac.uk/projects/fastqc/) v0.11.8. Quality trimming and adapter removal were done with fastq-mcf v1.05 tool using minimal quality of 5 bp window setting of 18 [13]. Overlapping paired-end reads were merged with SeqPrep (https://github.com/jstjohn/SeqPrep) v1.2. Metagenome was assembled with SPAdes assembler v3.14 in metagenomic mode [14].

Metagenomic binning was performed by nonlinear dimensionality reduction and density-based clustering of nucleotide composition and coverage depth data [15]. Shortly, large contigs were cut into 10,000 bp fragments and small contigs (less than 1000 bp) were excluded from the analysis. After that mean contig fragment coverage was assessed by mapping the reads with Bowtie2 v2.4.1 to contigs [16] and estimation of mean coverage with samtools v1.10 [17]. In parallel, frequencies of nucleotide tetramers were assessed for each fragment with custom Perl script. Then tetramer frequencies were combined with average fragment coverage to form a 257-dimensional array, which was processed with tSNE [18] dimensionality reduction approach to form a two-dimensional array. Reduced data was clustered with HDBSCAN tool v0.8.26 [19] and finally fragments of contigs included in the same cluster were merged back and metagenomic bins were formed.

The bins were manually curated by inspecting BLAST + v2.9 [20] alignments against available genomes of both cultured and uncultured *Thermoplasmatales* archaea. The quality of obtained bins was assessed using CheckM v1.1.2 [21].

### Bioinformatics analysis

Four different genomes were used for the comparison among *C. divulgatum* and “E-plasma”: *C. divulgatum* S5 (GCF_900083515.1), *C. divulgatum* PM4 (GCF_900090055.1), “E-plasma” from Iron Mt, USA (GCA_000496135.1) and “E-plasma” from Parys Mt. In the case of *C. divulgatum* genomes, genes and amino acid sequences were downloaded from the National Center for Biotechnology Information (NCBI) Genome database, as well as the existing genome assembly for the “E-plasma”, Iron Mt. For both “E-plasma” genomes (GCA_000496135.1 and data presented here), coding genes and amino acid sequences were predicted using Prokka v1.14.5 [22]. Orthologs among the four genomes were obtained using Roary v3.12.0 [23]. Additional functional annotation was performed with ec2KEGG v1 [24] in order to add KEGG annotation to genes with Prokka-annotated EC numbers. ArCOGs [25] database was also fetched from NCBI and used with psi-blast from BLAST + v2.10.0 [20] to annotate orthologs sequences between *C. divulgatum* and “E-plasma” genomes returned by Roary v3.12.0. “E-plasma” secretome was predicted using the SignalP 6.0 server [26]. Phylogenetic tree locating Parys Mt “E-plasma” bin was calculated using GTDB-Tk v2.1.1 with *de novo* workflow [27].

For ancestral reconstruction and pangenome analysis of *Thermoplasmatales* order the 22 GTDB species representative genomes of this taxon were downloaded from the NCBI using datasets package v13.43.2 (https://github.com/ncbi/datasets), including genomes of both *C. divulgatum* mentioned above and Iron Mt “E-plasma”. The Parys Mt “E-plasma” bin was also added to the dataset of genomes for the analysis. Quality and descriptive parameters of the assemblies were assessed for the 23 genomes using CheckM v1.2.0 [28]. The genome assigned to *Thermoplasmatales* archaeon UBA582 (GCA_002502705.1) was finally discarded from analysis due to low completeness (63.79%). The completeness of final dataset of 22 genomes was in the range between 79.60 and 99.59%, averaging 95.66% (19 out of 22 genomes showed > 90% of completeness). Contamination range was from 0 to 6.11% (averaging 0.79%).

Core pangenome proteins were calculated using get_homologues v15062022 [29]. This resulted in 14,419 protein clusters, only 52 out of 14,419 were considered as core proteins, assuming, that those proteins are presented in all of the analyzed genomes. Annotations of protein clusters of proteins with Pfam domains was performed using the script annotate_cluster.pl, within get_homologues package. KEGG annotation was performed using KofamScan v1.3.0 [30], while arCOGs were annotated using HMMER v3.2 [31], producing a profile of Hidden Markov Models (HMMs) for each cluster and comparing each profile against arCOGs database.

Phylogenetic tree based on core proteins was constructed using a concatenated multiple sequence alignment (MSA) of core protein clusters using the following procedure. Firstly, individual alignments of the core protein clusters have been performed with Mafft v7.427 [32] using L-INS-i algorithm. Individual MSAs were trimmed using ClipKIT v1.3.0 [33]. Then concatenated MSA was produced using Geneious Prime v2023.4.0 ((https://www.geneious.com). Finally, phylogenetic tree was built by maximum likelihood following a WAG + I + G substitution model, calculated by ModelTest, included within phangorn R package v2.10.0 [34], with a bootstrap of 1,000 replicates.

For the pangenome ancestral reconstruction, was used the program Count v10.04 [35], calculating each gene gain, loss, expansion or reduction probability by Posteriors analysis, based on phylogenetic birth-and-death model.

All bioinformatic procedures were performed under a Linux-based platform Ubuntu 20.04.3 LTS, using the High-Performance Supercomputing Cluster from Supercomputing Wales (SCW, https://www.supercomputing.wales/). Figures have been developed using R programming language v4.2.1 [36] using core, ape and the abovementioned phangorn packages.

## Results and discussion

### The general genome characteristics of “E-plasma” (Parys Mt)

The Parys Mt “E-plasma” bin showed a final genome size of 1.68 Mbp, with a 93.10% of completeness, 0.87% of contamination, a G + C content of 38.48% and a total number of 1,831 predicted coding genes. In comparison, the Iron Mt “E-plasma” had the genome size of 1.66 Mbp and 94.71% of completeness in 132 contigs (N50: 25,093).

### Genome-based phylogeny of “E-plasma”

**Fig. 1:**
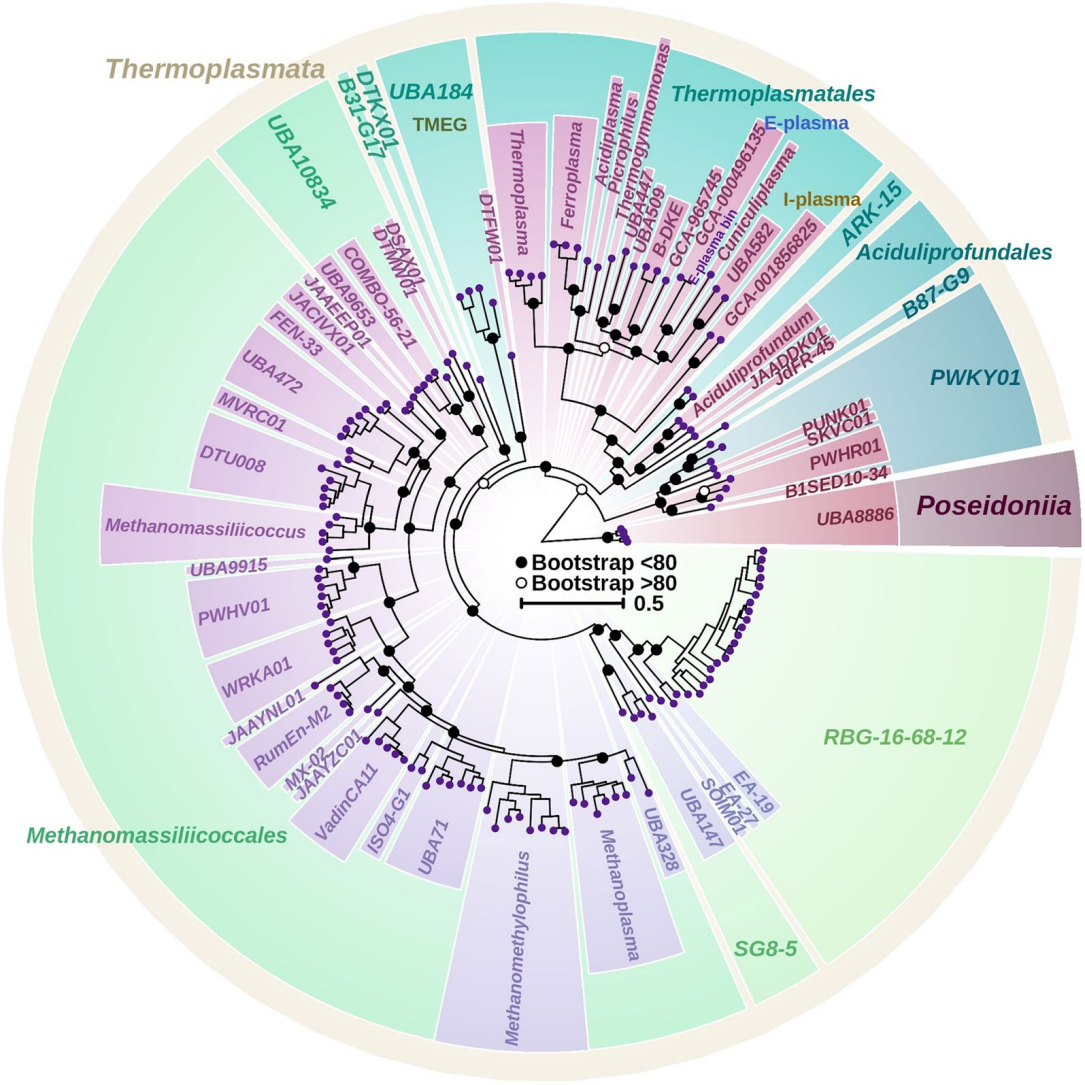
Phylogenetic tree based on 122 proteins from *Thermoplasmatota* archaea as per GTDB dataset. The tree was calculated using GTDB-Tk program to locate the genome within the *Ca*. Thermoplasmatota phylum, using the phylum *Ca.* Poseidoniia as the outgroup. Bootstrap results shown with open (< 80) and closed (> 80) circles. Parys Mt “E-plasma” bin is placed exactly next to the Iron Mt “E-plasma” genome GCA_000496135.1, within the order *Thermoplasmatales*. Colours used to distinguish between different archaeal clades

The phylogenetic tree based on 122 concatenated proteins firmly placed “E-plasma” into the order *Thermoplasmatales* (Fig. 1). It is also clear that the closest to “E-plasma” archaea were *C. divulgatum* since these organisms showed distinct clustering and branched relatively deeply from other members of the order. Of note, the 16S rRNA genes of “E-plasma” and *C. divulgatum* had 93% sequence identity, considering that based on this criterium organisms belong to one family within the order [37]. Another nearest organism to “E-plasma” was represented by the UBA582 metagenomic assembly (BioProject PRJNA348753) retrieved from biofilm metagenome from Richmond Mine C10 location, Iron Mt (USA) [38].

### Comparison of genomes of “E-plasma” Parys Mt to *C. divulgatum* and “E-plasma” variant from Iron Mt

**Fig. 2:**
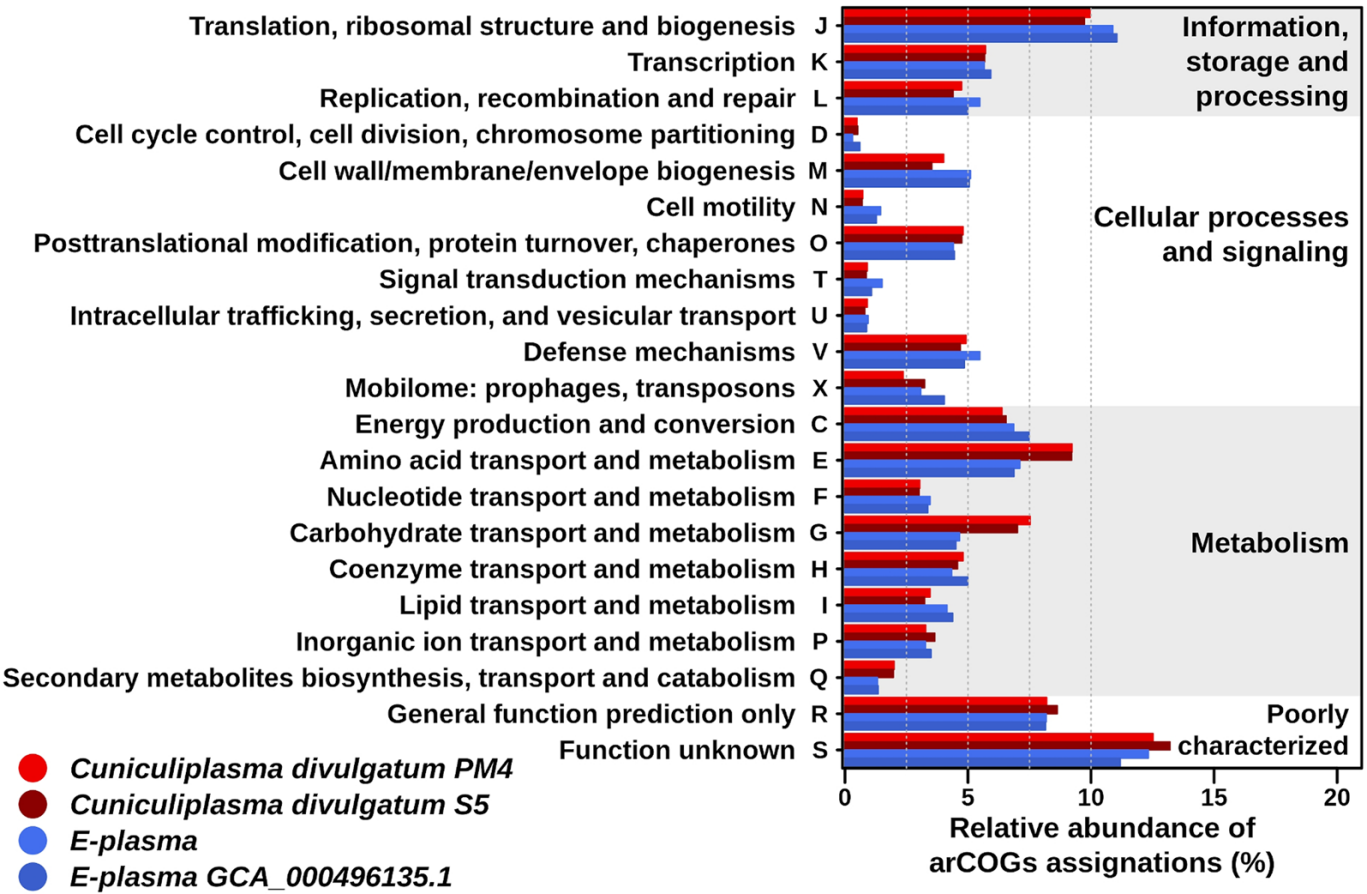
Functional categories of genes in four genomes. For the analysis were used genomes of both “E-plasma” variants (Parys Mt, UK and Iron Mt, USA) and of *C. divulgatum* strains PM4 and S5. Relative abundances of genes in genomes are shown according to functional arCOGs categories, %. Assignments are based on the results of psi-blast against arCOGs database using the pangenomic sequences returned by Roary v3.12.0. The relative abundance of arCOGs per genomes of both “E-plasma” are shown in blue colours, while *Cuniculiplasma* genomes are shown in red colours

To elucidate mechanisms underpinning the remarkable environmental fitness of “E-plasma”, we compared its genome with that of *C. divulgatum* PM4, previously isolated from Parys Mt, whose abundance in situ is 1–2% of the total microbial community [3, 39]. Both organisms coexist in the same environment, and therefore, genome comparison was suggested to be indicative for genomic signatures devoted to other metabolic pathways, or additional functions. Moreover, the genome of another *C. divulgatum* S5, isolated from very similar environment (copper mine stream, Cantareras mine, Spain) was included into the analysis. In parallel, the comparative analysis was conducted between genomes of “E-plasma” variants identified in Iron Mt (USA) and Parys Mt (UK), located on different continents (North America and British Isles/Eurasia, correspondingly). The analysis revealed a number of differences in the content of genes of functional categories (Fig. 2 and Additional file 1: Table S1).

The higher numbers of genes in genomes of “E-plasma” in comparison to *C. divulgatum* strains PM4 and S5 were affiliated with particular subcategories within all categories, i.e. “Replication, recombination and repair”, “Cell wall/membrane/envelope biogenesis”, “Cell motility”, “Defence mechanism”, and “Nucleotide transport and metabolism” (Additional file 2: Supplementary Information, Additional file 1: Table S1).

High percentages of genes associated with the category “Information, Storage and Processing” are reported to be characteristic for DPANN (Diapherotrites, Parvarchaeota, Aenigmarchaeota, Nanoarchaeota and Nanohaloarchaeota) organisms [40]. It was shown previously that community structure shaping genes (CSS) considered as crucial for structure of microbial representation are associated with “Mobilome and Cell motility” categories [41]. Another comparative genomic study involving bacterial genus *Acidiphilium* suggested an increase in genes of COG subcategories C (“Carbohydrate transport and metabolism”), L (“Replication, Recombination, and Repair”), P (“Inorganic ion transport and metabolism”), and N (“Cell motility”) as an attribute of adaptation to acidic conditions [42].

Similar numbers of genes affiliated with arCOGs of various functional subcategories were observed in both “E-plasma” genomes. Some insignificant discrepancies in those numbers are reflected in the Additional file 1: Table S1.

### Central metabolic pathways of “E-plasma”, Parys Mt

All enzymes involved into a tricarboxylic acid cycle (TCA) were identified in the genome of “E-plasma”. All gluconeogenesis and glycolysis-related genes were found, apart from the glucokinase (EC 2.7.1.2). Furthermore, enzymes potentially involved into a non-oxidative pentose phosphate pathway were identified. Nonphosphorylative Entner-Doudoroff pathway genes, including those for the key enzyme, KdgA, 2-keto-3-deoxygluconate aldolase, were found as well (Additional file 1: Table S1).

#### Glyoxylate shunt

**Fig. 3:**
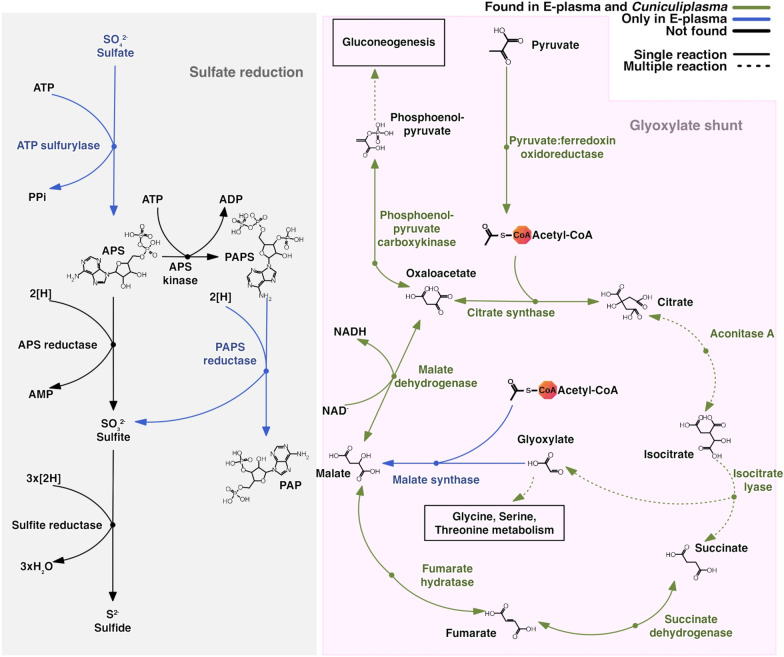
Metabolic reconstruction of selected pathways (Sulfate reduction and Glyoxylate shunt) for “E-plasma” and *Cuniculiplasma divulgatum*. Presence of relevant genes in both genomes are shown in green colour. Presence of encoding genes, only present in “E-plasma” shown in blue colour and the absence of genes in both genomes (“E-plasma” and *Cuniculipasma divulgatum*) shown in black colour. Steps including more than one reaction shown as dashed arrows

Similarly, to Iron Mt “E-plasma” genomic assembly, key enzymes for the glyoxylate shunt, isocitrate lyase and malate synthase were identified in its Parys Mt counterpart (Fig. 3). Of note, both proteins showed a high sequence identity (> 60%) with corresponding bacterial proteins and in the case of a malate synthase also to thaumarcheotal counterparts, pointing on possible lateral gene transfer origin for these genes. The glyoxylate shunt might enable the use of lipids for the synthesis of glucose or play role in oxidative stress response and thus, could be beneficial for environmental fitness of discussed organisms [12, 43]. However, the molecular mechanism underlying the link between oxidative stress and the glyoxylate shunt remains poorly understood, especially in archaea. According to [12], apart from “E-plasma”, other genomes of uncultured *Thermoplasmatales* from Iron Mt lack the genes for glyoxylate bypass.

Our analysis identified all five respiratory complexes in the “E-plasma” genome.

##### Iron oxidation potential

The iron oxidation represents a beneficial physiological trait for microorganisms residing in ferrous- and ferric-rich environments and was previously reported in archaea of the order *Thermoplasmatales* [44, 45]. A key gene for this process, a sulfocyanin was present in the “E-plasma” genome as well, with the highest amino acid sequence identity value (48%) to a cupredoxin-containing protein from *Thermogymnomonas acidicola* and with 40% sequence identity to a sulfocyanin of *C. divulgatum* S5. Of note, corresponding blue-copper protein-encoding gene was not found in the Iron Mt “E-plasma” genome [12]. In relation to that, in the *Thermoplasmatales* genomes these genes are quite often located on genomic islands, pointing at their transferability between organisms [3, 38, 46–48]. Supposedly, sulfocyanins might be involved into iron oxidation respiratory complex of “E-plasma” [47]. However, the presence of corresponding genes in a genome cannot per se serve as a direct hint to the iron oxidation: neither *Picrophilus torridus* or *C. divulgatum* encoding these proteins exhibited ferrous oxidation in experimental trials [3, 39, 46, 49]. The earlier experimental study of [50] showed that the isolated respiratory membrane protein complexes involved into iron oxidation in *F. acidiphilum* Y^T^ included (1) *aa*3-type cytochrome and a blue copper protein, and (2) a *ba* complex (di-heme cytochrome and a Rieske protein). It is also known that the genomes of *Thermoplasmatales* possess two respiratory complexes which might be connected with iron oxidation, a putative SoxM terminal oxidase and a *bc* complex [50]. Apart from a sulfocyanin gene we detected in the genome of “E-plasma” a polyprenyltransferase (cytochrome oxidase assembly factor), a heme/copper-type cytochrome/quinol oxidase, subunits 1, 2 and 3 and a cytochrome b subunit of the bc complex and Rieske Fe-S protein. To conclude, the genes for aerobic respiration were present in the “E-plasma” genome, which is consistent with their oxygen-dependent *modus vivendi*, however, the iron oxidation still needs to be experimentally validated in a pure culture.

##### Sulfur metabolism

The gene associated with the oxidation of sulfur to sulfite encoding the heterodisulfide reductase, subunit C (identity < 50% with DUF2440 family proteins from *Ferroplasma* and *Acidiplasma*) was present in the “E-plasma” genome. Interestingly, we found an ATP sulfurylase (identity < 50% with *Thermoprotei* archaea, *Caldisphaera* sp., *Ferroplasma* sp., *Methanocaldococcus* sp.) and a PAPS reductase (3′-phosphoadenosine 5′-phosphosulfate sulfotransferase) in the genomic assembly, known to catalyse two of four steps in assimilatory sulfate reduction in bacteria. Of note, these genes were absent in the *C. divulgatum* PM4 and S5 genomes (Fig. 3). Furthermore, genes for a cysteine desulfurase and cysteine desulfurase activator proteins, SufC and SufB, were present in two copies in the genome assembly.

##### Proteolytic proteins

A high proportion of genes encoding proteolytic proteins was revealed in the “E-plasma” genome. These proteins are in line with proteolytic/peptidolytic lifestyles of isolated and studied *Thermoplasmatales* [3]. According to [51, 52] those proteolytic proteins are also quite common in *Euryarchaeota* and it could also be used for carbon/energy metabolism. In more detail, we found subtilases of S53 superfamily and thermopsins, known to be associated with cell membrane or released into the extracellular milieu [51].

Subtilases, peptidase from A4 family, and thermopsin (the latter gene, represented in three copies) were identified among secreted proteins (Additional file 3: Table S2). In relation to that, the role of the subtilases counterparts in haloarchaea were shown to possess antagonistic and defensive abilities [50], which may also be the case for *Thermoplasmatales*. Among other proteasome systems, α- and β-types subunits of ATP-dependent 20S proteasomes, together with ATPase of the AAA+ class, CDC48 family and 20S proteasome assembly factor/ATP-grasp superfamily enzyme encoding genes were identified in the genome.

Furthermore, Lon proteases and serine proteases of S46 superfamily were detected, which are membrane-bound enzymes (Additional file 3: Table S2) hydrolysing extracellular proteins for their assimilation and are very common in *Thermoplasmatales* [51, 53, 54].

Among the proteolytic proteins, membrane-integral proteases used for translocation, such as type I signal peptidases of the S24, S26 LexA/signal peptidase superfamily, the A22 family presenilin-like membrane protease, A24 subfamily prepilin-like peptidases B, and site-2 proteases (S2P) class of zinc metalloproteases of MEROPS family M50 were identified in the “E-plasma” genome assembly. Genes encoding intracellular peptidases for amino acid recycling, namely type II methionine aminopeptidases, family 24 peptidases, L-asparaginase type 2-like enzymes of the NTN-hydrolase superfamily, P family Xaa-Pro aminopeptidases with a creatinase/prolidase N-terminal domain, gluzincin peptidase family (thermolysine like) aminopeptidase N, M1 family metallopeptidase and leucyl aminopeptidases were present in the genome as well.

Proteolytic proteins might be successfully employed by “E-plasma” for decomposing cells of community members. Organic carbon used by “E-plasma” could also originate from algae, co-inhabiting the site.

##### Transporters

Among transporters, encoded in the genome, the most significant fraction (47) being ABC transporters. Among ABC-type transporters Mn/Zn-, Fe^3+^-hydroxamate-, molybdate- and cobalamin/Fe^3+^ siderophores- transport related proteins were found. Of note, another ABC-type transport system, involved in multi-copper enzyme maturation, permease component, NosY found in many (7) copies in the genome. Furthermore, should be noted numerous presences of ABC-type multidrug transport system component, CcmA. Also, ABC-type anion- and nitrate/sulfonate/bicarbonate transport system components were identified.

A subset of amino acids transporters and oligo/dipeptides transporters (7 and 4, respectively) was noticed to be present in the “E-plasma” genome too.

##### Glycosyl hydrolases

The genome of “E-plasma” harbours glycoside hydrolases (GHases) of various GH families, including GH13, GH15, GH18 and GH31 as per CAZY database [55]. GH15 of “E-plasma” showed highest identity to GH15 proteins from other *Thermoplasmatales*. Biochemically studied activity of GH15 was confirmed as a glucoamylase in *Thermoplasmatales* archaeon *P. torridus* and as a trehalase in *Thermoplasma acidophilum* and *T. volcanium* [56, 57]. Another “E-plasma” glycosyl hydrolase GH31 family, showed 52% identity with *C. divulgatum* homologue and 42% amino acid sequence identity with functionally characterised alpha-glucosidase/maltase from *Saccharolobus (Sulfolobus) solfataricus* P2 [58] and, albeit less similar, with a-glucosidases from *P. torridus* and *T. acidophilum* previously [59, 60]. Glycogen debranching enzyme (GH13) had its top homologues in “A-plasma” and *C. divulgatum* with 67 and 62% AA sequence identity, correspondingly, and 52% with functionally characterised counterpart of *S. solfataricus* P2 [61]. Family GH15 protein had its top homologues in predicted *C. divulgatum* protein (48% sequence identity) and, among functionally characterised proteins, to the clostridial 1,4-alpha-glucosidase (37%) [62].

##### Metal resistance and uptake

Genetic loci determining resistance to heavy metals were previously identified in the genomes of *Thermoplasmatales* and were mostly localised within genomic islands [39, 47]. Accordingly, the PM4 site on Parys Mt is characterised by high concentrations of metals and metalloids, including arsenic [4]. Our search for metal resistance genes in an “E-plasma” genome has identified several copies of arsenic efflux pump-related gene cassettes for ArsB and ArsR. Additionally, a putative arsenate reductase and a chromate resistance protein (arCOG10427), a putative copper-exporting P-type ATPase TRASH/YHS-like protein, a metallochaperone and a Zn-dependent protease with chaperone function were identified in the genome as well. We cannot exclude other, yet unknown, metal resistance systems in this organism.

Of note, no mercury- or copper-resistance genes were previously found in archaeal metagenomic assemblies from Iron Mt, AMD site that also included “E-plasma” variant [12].

Among metal transport systems identified in the “E-plasma” genome, an ABC-type transport system for Mn/Zn, a Fe^3+^-hydroxamate, cobalamin/Fe^3+^-siderophores, a dipeptide/oligopeptide/nickel, molybdate transport and a predicted divalent heavy-metal cations transporter of arCOG00576 were identified.

##### Oxidative stress

Oxidative stress management proteins were found in the “E-plasma” genome in line with its likely aerobic lifestyle. Genes encoding a peroxiredoxin, a thioredoxin, a catalase-peroxidase, a rubrerythrin and a superoxide dismutase were identified. Furthermore, a spermidine synthase involved into formation of spermidine from putrescine was detected in the genome, which together with trehalose might provide an osmo-protective mechanism. The synthesis of trehalose was supported by identification of genes encoding for a trehalose-6-phosphate synthase and a trehalose-6-phosphate phosphatase associated with a trehalose biosynthesis pathway TPS/TPP. Additionally, two genes, encoding aquaporin/glycerol uptake facilitator AqpM involved into osmoregulation were detected. In relation to an aquaporin, it is worth noting that these genes were only present in *Thermoplasmatales* from AMD: *C. divulgatum*, B_DKE strain (“*Scheffleriplasma*”) and *Ca*. Thermoplasmatota assembled from acidic mine tailings, China. It was suggested a proton-blocking function of these proteins in extreme acidophiles [63].

##### Secretome

Altogether, 55 proteins belonging to different categories were predicted to be secreted (Additional file 3: Table S2). High proportion among secreted proteins (23) were hypothetical or uncharacterised proteins that did not match arCOGs.

Of note, a pleckstrin domain containing protein (arCOG06115) was identified as secreted in an “E-plasma” genome. This gene was in a close proximity to KaiC family ATPase (arCOG01171) implicated in signal transduction, to a PIN domain containing protein belonging to a large nuclease superfamily (arCOG02730) and to cytotoxic translational repressor of toxin-antitoxin stability system RelE and Phd family antitoxin. It should be added that no genes encoding pleckstin domain-containing proteins were detected in *C. divulgatum*, PM4. However, this protein, is also present in Iron Mt “E-plasma” genome (but not in other *Thermoplasmatales*), and probably is a molecular signature for this group of archaea. Although, the antiSMASH (https://antismash-db.secondarymetabolites.org/) and BACTIBASE (http://bactibase.hammamilab.org/main.php) databases search did not return any genomic loci for secondary metabolites or bacteriocins in the “E-plasma” genome, we consider that biochemical characterisation is needed in the future to understand the function of a pleckstrin homology domain containing protein. It is known that those proteins are instrumental in the transport of enterocin [64], as bacteriocins production might be a significant trait contributing to the environmental success for these archaea. Prokaryotes also use peptides as communication signals in the-cross talk with other microorganisms [65]. Of note, another gene encoding an uncharacterized membrane protein YdbS, containing bPH2, arCOG04622 (bacterial pleckstrin homologue) domain with no similarity was identified in the “E-plasma” genome.

##### Cell surface structures

In the “E-plasma” genome, genes encoding archaellum proteins were co-clustered: Arl or FlaB, FlaC, FlaD, FlaF, FlaG, FlaH, FlaI, and FlaJ. Additional copies of genes for FlaI and FlaF, of the gene encoding a transcriptional activator of archaellum containing HTH domain, and for a signal transduction regulator, CheY, which contains Rec and HTH domains, were found as well. Considering the proteins FlaJ, FlaI and FlaH are known to be central for the archaellum motor operation, and chemotaxis protein CheY encoding gene were shown present too, we assume that “E-plasma” cells is likely flagellated and motile [66]. The presence of archaella but not chemotaxis encoding genes were noted for Iron Mt “E-plasma” genome [12]. It should be added that the presence of archaella and motility play important role in competition with other microorganisms via provision of significant advantage over nonmotile community members in attaining the supportive environment.

Genes FlaI, FlaG/FlaF and TadC/pilus assembly protein, represented in three copies might be an indication also of pili production by “E-plasma”. CAAX family protease associated with the type IV pili like system and a VirB4 component of type IV secretory pathway were also found in the genome. Furthermore, the genome possesses other genes associated with secretion system: an AAA+ ATPase of a MoxR-like family, a component of a putative secretion system, a Sec59, a preprotein translocase subunit SecD and SecY, a preprotein translocase subunit Sec61beta, a signal recognition particle 19 kDa protein, an OxaA/SpoJ/YidC translocase/secretase and wider elements. We identified also Sec-independent protein secretion pathway components twin-arginine translocases TatA and TatC with high levels of identity to counterparts from *Thermoplasmatales*. Of note, FlaF (two copies), FlaG and the number of cell surface proteins shown to be secreted (Additional file 3: Table S2).

The genome encodes a secreted protein with a beta-propeller repeat domain (arCOG02560 and arCOG02562), and a beta-propeller repeat protein fused to CARDB-like adhesion module, arCOG02532 (Additional file 3: Table S2). Sequences of both proteins with beta-propeller repeat domains showed identity (91%) to Iron Mt “E-plasma” and to some other YncE family proteins from uncultured *Thermoplasmatota*, which might be involved into intercellular interactions [67].

Proteins in the “E-plasma” genome associated with a cell wall/membrane/cell envelope biogenesis genes encoding a dTDP-4-dehydrorhamnose 3,5-epimerase, a RfbA/dTDP-glucose pyrophosphorylase, and a RfbD/ dTDP-4-dehydrorhamnose reductase might be involved into the L-rhamnose biosynthesis, which is known to be present in archaeal cytoplasmic membrane [68]. Also, multiple genes encoding nucleoside-diphosphate sugar epimerases might be involved into a cell wall/membrane/cell envelope biogenesis. Additionally, genes for nucleoside-diphosphate-sugar pyrophosphatases associated with lipopolysaccharides biosynthesis and cell adhesion were found.

Considering the presence in the genome of genes for Agl glycosyltransferases (AglB, AglD, AglF and AglR), with some of those genes present in multiple copies, one can suggest the cell surface of “E-plasma” may be covered by polysaccharides: Agl glycosyl transferases were shown to be involved into the glycan assembly pathways [69]. Of note, several cell wall/membrane biogenesis proteins of Parys Mt “E-plasma” had a rather low identity to proteins from bacteria or archaea, such as *Sulfolobales*, methanogens or “*Ca*. Bathyarchaeaota” but not to *Thermoplasmatales* proteins. Corresponding genomic loci had adjacent sequences of transposases and flanking insertion elements pointing at their potential origin via the lateral gene transfer.

We further identified an actin-like ATPase involved into cell morphogenesis, possessing conserved domains for plasmid segregation protein ParM and similar proteins. Of note, *T. acidophilum* Ta0583, an actin ATPase was not related to shape determination, but in chromosome separation and plasmid partitioning [70, 71].

##### Defence systems

The “E-plasma” genome harboured one array of 37 CRISPR (Clusters of Regularly Interspaced Short Palindromic Repeats), with 36 spacers. The genome contained genes for the subtype I-U of CRISPR-Cas, which was recently reclassified as a subtype I-G [69]. Subtype I-G contains a fusion of Cas4 and Cas1, which is the case in “E-plasma” genome. Genes for Cas2 (arCOG04194), CRISPR-associated RecB family exonuclease Cas4/CRISPR-associated protein Cas1 (arCOG00786), CRISPR-associated protein Csb1, an effector complex Csx17, and CRISPR-associated helicase Cas3 (arCOG01444) were identified. Additionally, the genes for Cas8 (arCOG10429), Cas1 (arCOG01452), Cas4-nuclease, Cas helicase and Casposon-associated uncharacterised protein (arCOG06521) were identified in the genome. The best hits among CRISPR-Cas associated proteins were found with those from “E-plasma” counterpart from Iron Mt. With other archaea, the amino acid sequence identity above 50% was shown with deduced *Thermoplasmata* proteins from deep terrestrial subsurface sediment metagenomes (BioProject PRJNA321556) and hot spring of Yellowstone National Park, USA (BioProject PRJNA480137). Also, CRISPR-Cas associated proteins from “E-plasma” had sequence identity above 50% with bacterial counterparts from *Clostridiales* (aquatic metagenome, BioProject PRJNA417962), and *Chloroflexi, Bacteriodaceae* and *Nitrospirae* (hot spring Yellowstone National Park, USA, BioProject PRJNA480137).

Of note, altogether 8 CRISPR-Cas associated genes were revealed in *C. divulgatum* PM4 genome [39]. *C. divulgatum* PM4 Cas proteins, namely Cas8b, Cas3, Cas4, Cas1, and Cas2 were also found in “E-plasma”.

Toxin-antitoxin systems (altogether 23 genes) were identified to belong to families VapBC, RelE/F, MazF, HicB and abortive phage infection protein AbiEi, antitoxin component of an AbiEi-AbiEii TA system. The latter were placed near a nucleotidyltransferase, a component of putative viral defence system, with the significant proportion of these genes being co-localised. Furthermore, genes encoding site-specific integrases-resolvases (arCOG03164 and arCOG01241/2, XeD/XerC family) and transposases (arCOG03965, arCOG02127, arCOG03473, arCOG02759, arCOG00684, arCOG04913, arCOG03585, arCOG10339, and arCOG04792), which are also a part of a Mobilome category were detected in the genome. Just of note, these genes showed homology to “E-plasma” Iron Mt phylotype, Parys Mt archaeal community members (*C. divulgatum, Ferroplasma* spp, “*Ca.* Micrarchaeum acidiphilum”) and to organisms without taxonomic status such as “A-plasma” and “I-plasma”.

It should be noted that the *C. divulgatum* PM4 genome possesses 32 genes associated with toxin-antitoxin systems [39]. The “Defence mechanisms” category was also previously shown being as significant fraction by *C. divulgatum* PM4, in comparison to *C. divulgatum* S5 from Cantareras, Spain AMD site, and to “G-plasma” metagenomic assembly from Iron Mt [39]. This might point at a significant pool of viruses in Parys Mt acidic ecosystem targeting different archaeal community members.

Also, various mobile element proteins and transposases were detected in the “E-plasma” genome.

We identified Type I restriction-modification system methyltransferase subunit, HsdM, restriction endonuclease S subunit, HsdS, type I/II restriction enzyme, methylase subunit and located nearly an ATPase, AAA+ superfamily fused to HTH and PD-(DE)xK endonuclease domains, known to be involved in the defence system in archaea [72, 73]. Important to note, that the type I restriction-modification system was not found in *C. divulgatum*, PM4 and Iron Mt “E-plasma” assembly, but nonetheless present in some *Thermoplasmatales* genomes (http://rebase.neb.com/rebase/arcbaclistA.html). However, Type II (R and M genes in multiple copies), Type II and Type IV genes were identified in the *C. divulgatum* PM 4 genome with altogether 14 genes associated with restriction-modification systems. Of note, only Type II R and M genes (4 genes in total) were detected in the Iron Mt “E-plasma” genome.

Genes for endonucleases III, IV, V in two copies, RecB family restriction endonuclease, SeqA-like protein, Endonuclease Nob1 and MvaI/BcnI restriction endonuclease family protein were present in the “E-plasma” genome as well.

Regarding the “E-plasma” mobilome, a nucleotidyltransferase, a component of viral defence system (arCOG03839), and a viral packaging ATPase, an AAA+ superfamily (typical for *Fuselloviridae*) (arCOG02242) were revealed. Interesting to note here, that viruses of families *Fuselloviridae* were identified in the domain Archaea only and often observed in extreme environments [74]. Additionally, two bacteriophage protein gp37-encoding genes were determined in genomic loci, enriched in hypothetical proteins. Bacteriophage proteins gp37 showed identity (> 40%) to *Microcoleus* sp. (Terrabacteria group) and to *Spirochaetaceae* bacteria. Moreover, genes encoding for a casposon-associated protein arCOG06521, a RepA plasmid replication protein, arCOG08578 and a phage or plasmid associated DNA primase, a primpol domain, arCOG06914 were identified in the genome as well.

### Evolutionary trajectories of “E-plasma” and other *Thermoplasmatales*

**Fig. 4:**
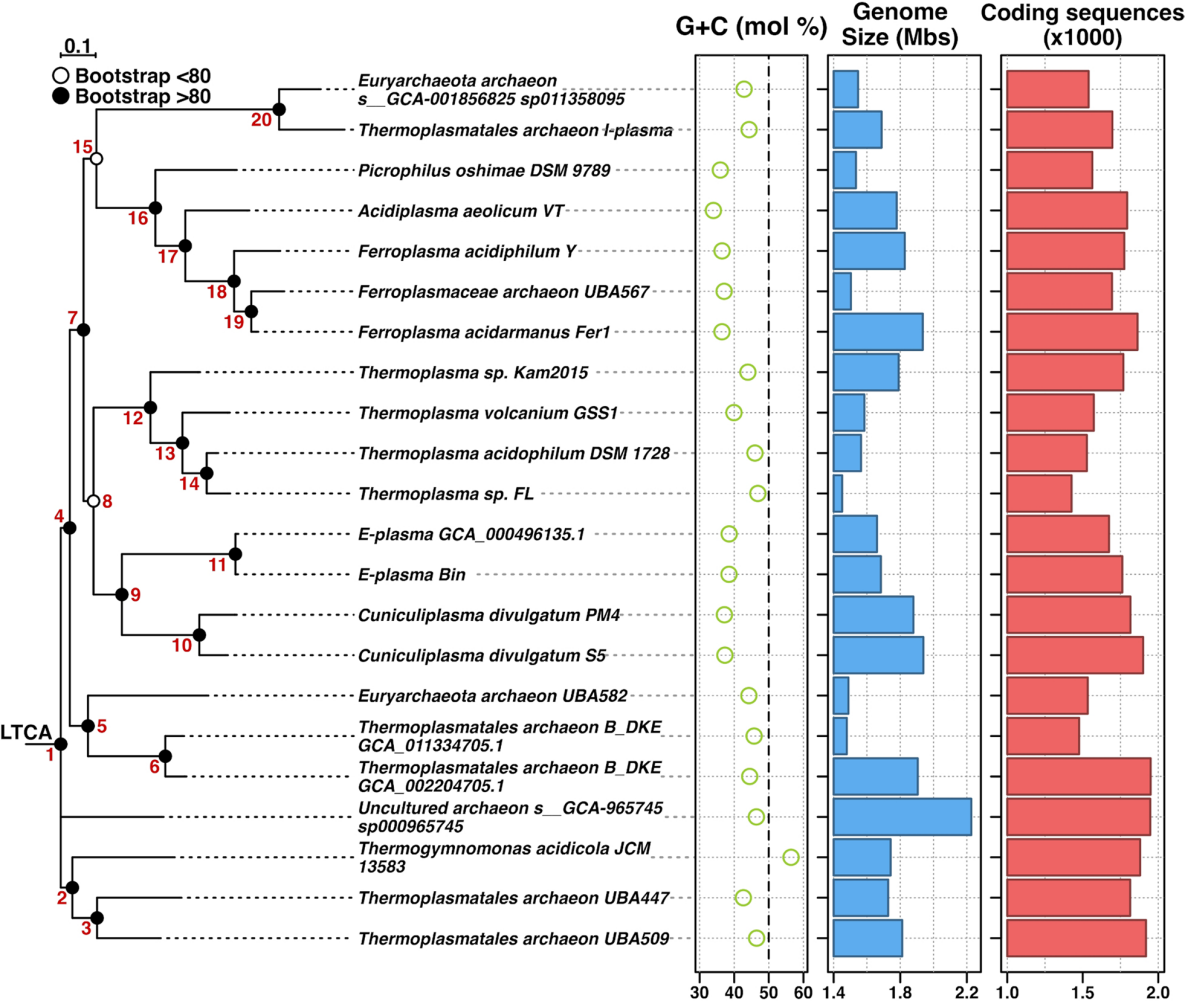
*Thermoplasmatales *phylogenetic tree based on 122 concatenated protein sequences in combination with other data. 122 concatenated protein sequences combined with: G + C mol%, Genome size (Mbp) and Coding sequences (x1000). Bootstrap results shown with open (< 80) and closed (> 80) circles

The evolutionary patterns of “E-plasma” and other archaea constituting the same order suggest that at the very early stages (Node 1, Fig. 4), the common *Thermoplasmatales* ancestor gave a rise of two distinct lineages, one including *T. acidicola* with two accompanying archaeal lineages represented by MAGs UBA509 and UBA447, and the second, including all other known genera of *Thermoplasmatales* with validly published names. *T. acidicola* is a clear outlier also in relation to its very high G + C mol content in the DNA (56.4%), while in all other *Thermoplasmatales* this value ranges between 34 and 46%. Its placement suggests that *T. acidicola* being the closest to the Last *Thermoplasmatales* Common Ancestor (LTCA) in terms of its metabolism among all cultured members (Fig. 4). In relation to the motility of *T. acidicola*, should be added that no archaella were visualised in this organism under tested growth conditions [75]. However, its genome encodes all homologues for archaella biogenesis, including the motor components (FlaH, FlaC, FlaD/E, FlaI), membrane-associated FlaJ, outermembrane-associated FlaF and flagellins. All these proteins have a high protein sequence identity (> 50%) with their counterparts from *T. acidophilum*, where archaella are produced, and where most of genes for these proteins are co-localised in one gene cluster, locus tags Ta0553-Ta0560 (GenBank Acc. Nr. NC_002578.1). This may imply that LTCA could have a genomic potential for production of archaella (genes encoding proteins *FlaC, FlaD, FlaG, FlaH, FlaI* and *FlaJ* were present on the LTCA). All other *Thermoplasmatales* formed a separate cluster at the branching point 4, from where two lineages evolved at the Nodes 5 and 7. The Node 5 ancestor gave a rise to a clade including B_DKE and *Thermoplasmatales* archaeon UBA582. Of note, according to this partition, B_DKE had a relatively distinct evolutionary trajectory among *Thermoplasmatales* inhabiting AMD systems. From Node 7, a large cluster of organisms including all cultured species with validly published names, except *T. acidicola*, was formed, with a monophyletic, single-genus, *Thermoplasma* branch (from the node 12), the cluster including genera *Picrophilus* and ferrous-iron oxidising organisms *Ferroplasma* and *Acidiplasma* (node 18), *Cuniculiplasma-*“E-plasma” cluster (at the node 9) and “I-plasma”-GCA_0018568095 (node 20) (Fig. 4 and Additional file 4: Table S3).

#### Gene gains and losses

Analysis of gene gains in “E-plasma” showed the acquisition of counterparts for signal transduction e.g. for histidine kinase ATPase domain protein, a phosphate starvation-inducible protein PhoH, a predicted ATPase, a serine/threonine protein phosphatase PP2A family, a membrane-associated serine/threonine protein kinase, which might be beneficial for environmental response of this archaeon. Further acquired genes encode key enzymes for the glyoxylate shunt, malate synthase and isocitrate lyase, which were found exclusively in both “E-plasma” genomes (Parys and Iron Mt).

**Fig. 5:**
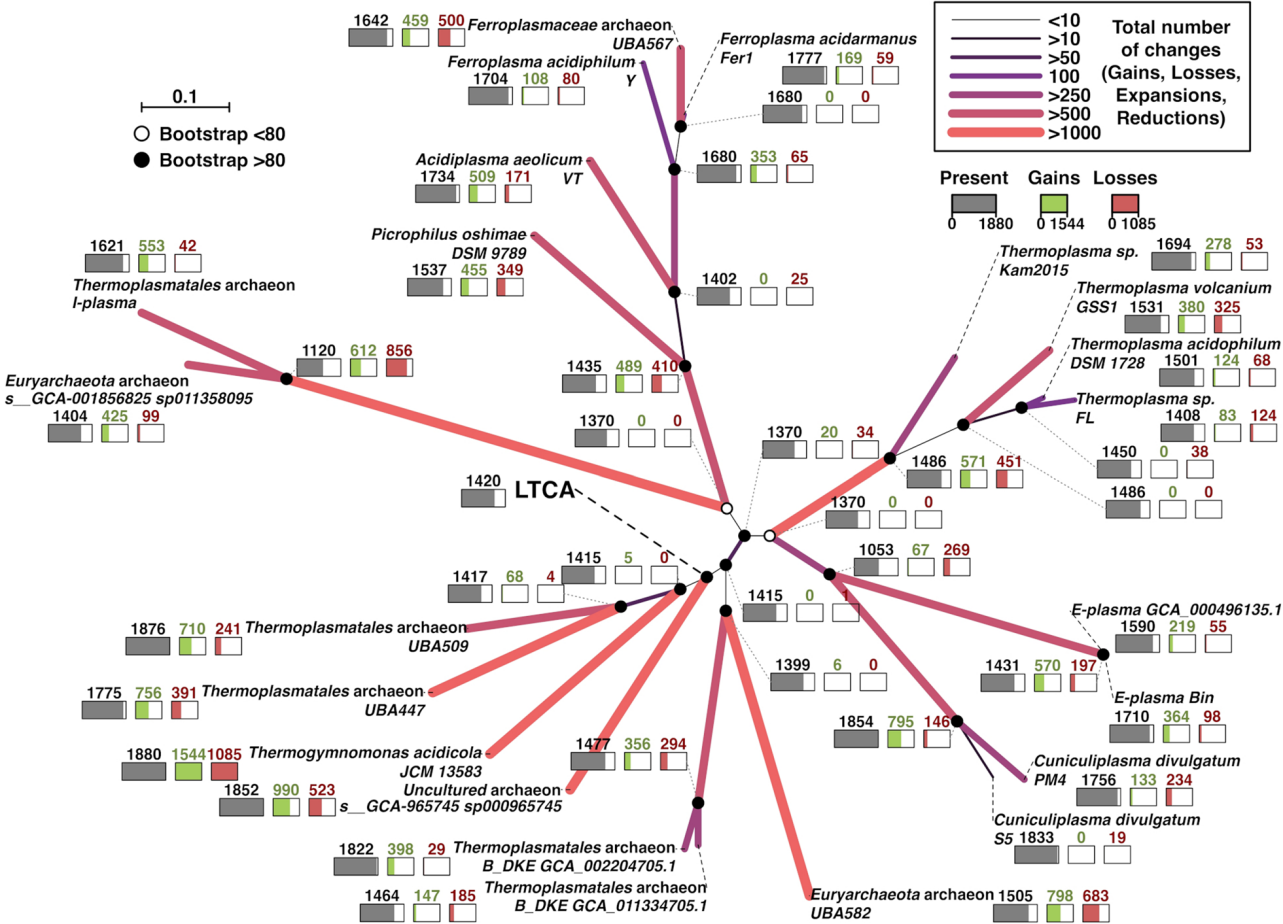
Unrooted core protein families-based phylogenetic tree of *Thermoplasmatales* illustrating gene gains/losses. Bootstrap values are shown as open (< 80) and closed (> 80) circles. Total numbers of modifications (sums of gains, losses, reductions and expansions) are shown with lines of different thickness and colour (see the code in the top right corner of the figure). At each node, the numbers of genes present in ancestors (grey), gene gains (green) and losses (red) are shown. Bars of similar colours below those numbers represent relative proportions vs. the maximum number of genes among all ancestors, e.g. 1.854 genes present and 795 gene gains in *Cuniculiplasma* ancestor, or 856 gene losses, shown for “I-plasma” ancestor

Other genes gained by the “E-plasma” genome were an ATP sulfurylase and a CysH 3′-phosphoadenosine 5′-phosphosulfate sulfotransferase (PAPS reductase)/FAD synthetase or related enzyme. However, it is currently unclear whether these genes might be functional for this archaeon. It should be noted that these genes were not detected in ancestors of the ”E-plasma”-*Cuniculiplasma* branch or in LTCA (Fig. 5, Additional files 5 and 6: Tables S4 and S5).

Some cell wall/membrane/envelope and cell motility categories of genes were acquired by “E-plasma”: a nucleoside-diphosphate-sugar epimerase, an UDP-N-acetyl-D-mannosaminuronate dehydrogenase, predicted pyridoxal phosphate-dependent enzyme apparently involved in regulation of cell wall biogenesis and archaellum-associated genes were present in lower copy numbers or were absent in *C. divulgatum* PM4 in contrast to “E-plasma”. It might be pointed out that that according to this analysis, the “E-plasma”-*Cuniculiplasma* branch ancestor, “E-plasma”, all *Thermoplasma* spp. from the neighbouring branch, which rose from the node 12, very likely *T. acidicola* and possibly the LTCA had archaella as well, while all taxa originating from the node 15 (*Picrophilus-Ferroplasma-Acidiplasma-*“I-plasma”) have lost the ability for forming archaella.

**Fig. 6:**
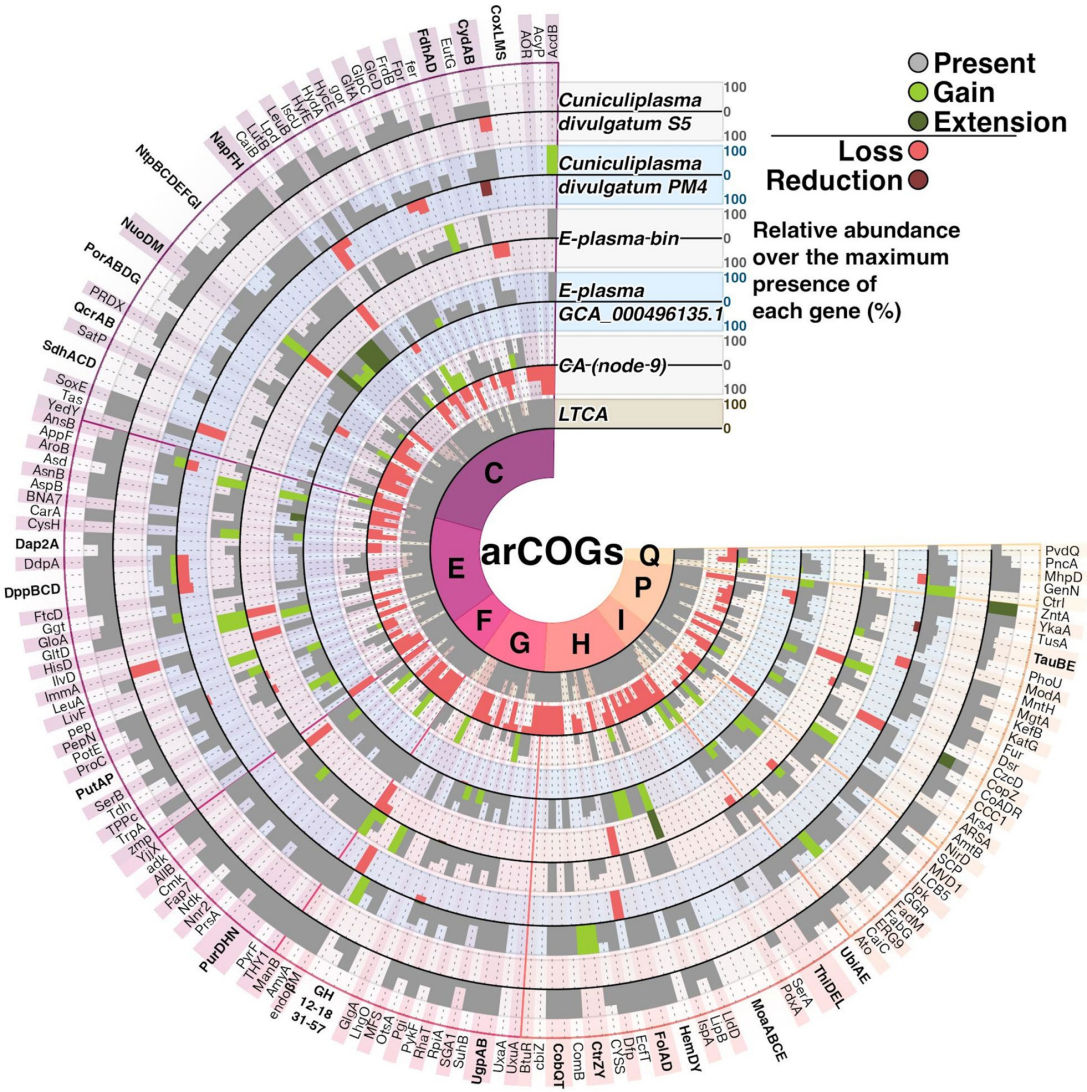
The census of proteins from metabolism-related subcategories of arCOGs in “E-plasma”-*Cuniculiplasm*a core protein families. Bar sizes of 100% correspond to maximal numbers of each gene along the genomes and ancestors included in the branch. Bold labels correspond to proteins encoded by an operon, or multidomain complexes. For each protein, grey bars represent the number of genes in a genome. Overlapping grey bars of sizes corresponding to the percentage of present gene numbers to gains (light green) or expansions (dark green). Bars opposing the grey bars show relative numbers of losses in that genome for each gene. CA, the Common Ancestor of the “E-plasma”-*Cuniculiplasma* branch (the Node 9 in Fig. 4; LTCA, Last *Thermoplasmatales* Common Ancestor)

Other genes, present in several copies in “E-plasma” in comparison to gene numbers in PM4 strain and in both ancestors (”E-plasma”-*Cuniculiplasma* clade and in LTCA) were coding for an AglL, a glycosyltransferase, transposases and other proteins from subcategories C, M and X (Figs. 5, 6, Additional files 5 and 6: Tables S4 and S5). L subcategory genes for a DNA repair photolyase and an UvrD, superfamily I DNA and RNA helicase were gained as well (Figs. 5 and 6, Tables S4 and S5).

In relation to gene losses in “E-plasma” genome, genes for a cytochrome *bd*-type quinol oxidase, subunits I and II alongside with a HerA-like helicase, fused to PD-(DE)xK superfamily endonuclease, a superfamily II DNA/RNA helicase, SNF2 family, some further from “Defence mechanisms” subcategory were found missing (Fig. 6 and Additional files 5 and 6: Tables S4 and S5).

**Fig. 7:**
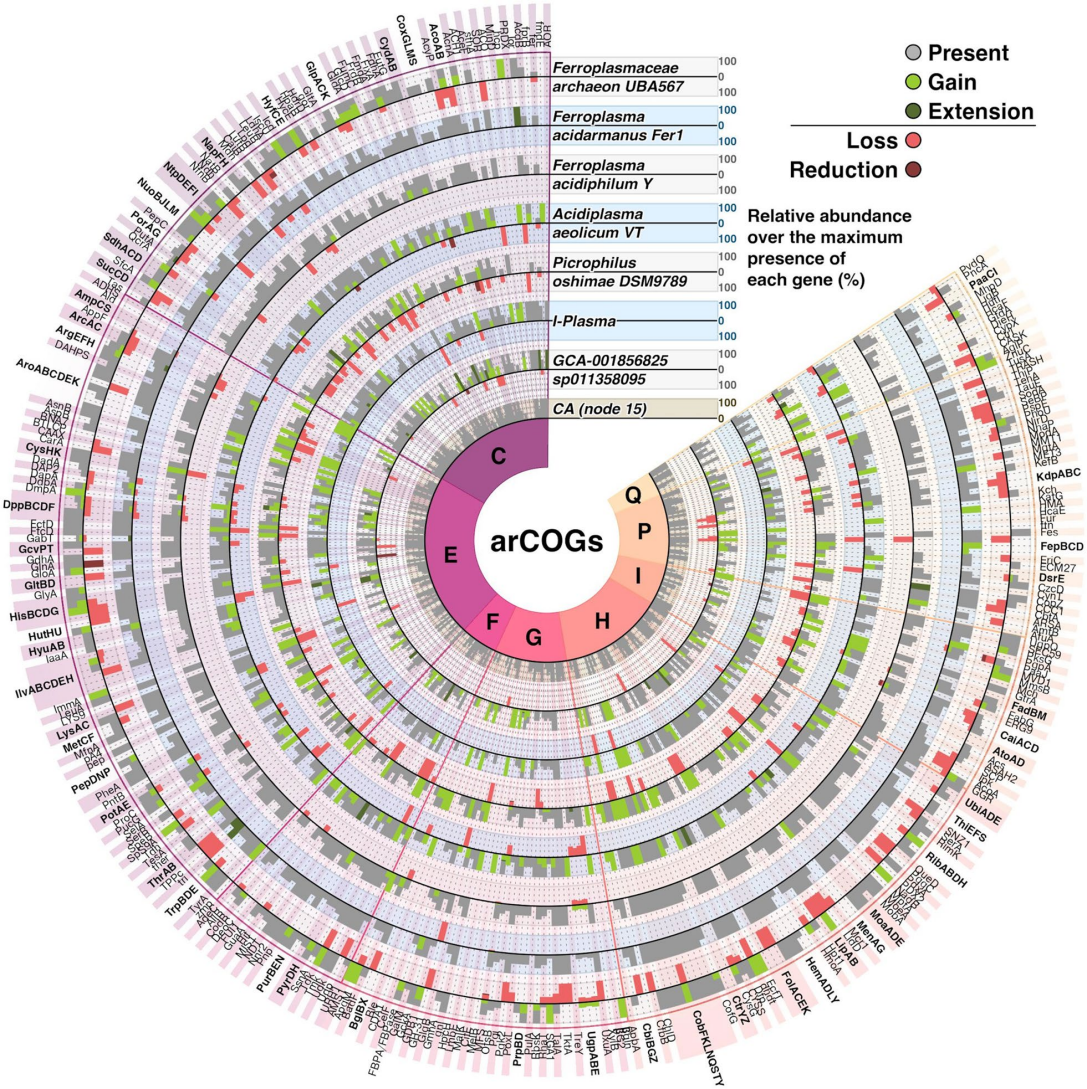
The pangenome of the *Ferroplasma-Acidiplasma-Picrophilus*—”I-plasma” cluster. The plot was drawn using metabolism related census of subcategories of arCOGs. Bars 100% the total number of modifications for each gene along the genomes/ancestors included in the branch. Bold labels indicate proteins encoded by an operon, or multidomain complexes. Grey bars show genes present in the genome. Gains are shown as light green, expansions as dark green, the gene losses are shown as red bars. CA, the Common Ancestor of the *Ferroplasma-Acidiplasma-Picrophilus*-”I-plasma” cluster. Please note that the organism/genome represented by each outer circle is not considered as a descendant of the neighbouring organism/genome, represented by inner circle

In relation to *Ferroplasma-Acidiplasma-Picrophilus*-”I-plasma” cluster, it must be noted that contrary to *Ferroplasma* and *Acidiplasma*, *Picrophilus* spp. experienced significant events of gene losses (Figs. 5 and 7, Additional files 5 and 6: Tables S4 and S5). Two main confirmed traits differ *Ferroplasma* and *Acidiplasma* spp. from *Picrophilus* spp., which are the acquisition of iron-oxidising capabilities in the former two organisms, and the presence of S-layer in the latter archaeon. However, genes associated with iron oxidation, such as cytochrome *bd*-type quinol oxidase, subunits I and II, cytochrome *b* subunit of the *bc* complex and sulfocyanin were found in all genomes of this cluster, common ancestor of this cluster, and in the LTCA.

In relation to counterparts, which might be linked to the formation of the S-layer, only a gene for a putative S-layer domain protein (COG1361) was predicted in *Picrophilus* spp., but also in *Ferroplasma-Acidiplasma* group and also in *Thermoplasma* spp. Whatever the case, as only in *Picrophilus* spp. S-layers were visualised by microscopy, one can hypothesize that the corresponding phenotype is linked with yet unidentified proteins that have been acquired by *Picrophilus* spp. in a recent gene transfer event. Other cell wall/membrane genes which might be mentioned here is an actin-like ATPase from the cell morphogenesis category found in cell wall-deficient *Ferroplasma, Acidiplasma* and their ancestors but not in the genome of *Picrophilus*. Actin in *Thermoplasma* was discussed previously and considered to be phylogenetically more closely related to proteins of ParM family [76].

Among other genes important for fitness of *Ferroplasma, Acidiplasma* and *Picrophilus* a DNA repair photolyase could be mentioned, which was predicted to be gained in all these organisms and was absent in both ancestors. Of note, proteins associated with formation of archaealla were present in both ancestors but were not present in *Ferroplasma, Acidiplasma* and *Picrophilus*, which was also confirmed by microscopy data [44, 45, 49], suggesting the loss of archaealla in this cluster of *Thermoplasmatales* as a general trend.

To summarise, “E-plasma” experienced major horizontal gene transfer events, which might have contributed to its success in AMD environment, despite its streamlined genome. Streamlining was suggested to be successful optimisation strategy for acidophilic bacteria [77]. Not to mention, that the neighbouring to *Cuniculiplasma*-“E-plasma” cluster includes only thermophiles of *Thermoplasma* spp. *T. acidicola* is risen from, and is situated closely to the, first branching point at the root of *Thermoplasmatales*, though its genome illustrated gains and losses in comparison to LCTA. This could support the hypothesis that *T. acidicola* may be an organism, which resembles the relic form of *Thermoplasmatales*, to a greater extent than other isolated and studied archaea of this order. Based on the phenotypic traits of *T. acidicola* of [75], LTCA archaea were probably occupying geothermal sites and were cell wall-lacking, thermophilic, aerobic, chemoorganotrophic, and rather moderately acidophilic organisms with a high molar G + C content, and with relatively small genomes. The events of gene gains and losses have led in some descendants to the extended metabolic versatility (e.g. iron oxidation, or facultatively anaerobic lifestyles), formation of a S-layer and to the global expansion of these organisms in the new acidic, non-thermal environments.

## Conclusion

The genome analysis supports the proteolytic/peptidolytic lifestyle of “E-plasma” similarly to all members of *Thermoplasmatales*. There was no clear indication of particular metabolic pathway, or any conclusively predicted metabolic trait which might provide a solid reason for predominance of ”E-plasma” in situ. Nonetheless, few discrepancies between gene content of a numerically predominant “E-plasma” and their counterpart sharing the same niche, *C. divulgatum* PM4, were observed. Foremost, the motility (presence of archaella) may have been an advantageous factor for predominance of “E-plasma”. In line with the genomic analysis, overrepresentation of genes from arCOG subcategories C, L, M and N in the “E-plasma” genome might be beneficial for the environmental fitness. A further isolation of its pure culture and omics studies will define the nature of competitiveness of these archaea in situ.

The more recent evolutionary history of “E-plasma” points that they form a cluster with *Cuniculiplasma* spp., both being very successful globally in the colonisation of the non-thermal AMD environments. Thermophilic counterparts from the lineage that included all known *Thermoplasma* spp., have been split from *Cuniculiplasma-*“E-plasma” group at an earlier timepoint. The further evolution within the *Cuniculiplasma*-“E-plasma” cluster resulted in their distinct genome contents and sizes, with the larger genomes in the former, and more streamlined in the latter. Nevertheless, despite their genomes have some 200 lower gene counts (or 12% less genes than *Cuniculiplasma* species) “E-plasma” significantly, ca. 30-fold, outnumbers *Cuniculiplasma* spp. in the same environment.

The ancestral reconstruction of *Thermoplasmatales* archaea reflects complex headways of these organisms, expansions and reductions in genomes and placed *T. acidicola* at the closest position to the root for this group among other cultured and studied members of the order.

## Supplementary Information

**Additional file 1. Supplementary Table S1.** Comparison of genomes of "E-plasma" Parys Mt to *C. divulgatum* and "E-plasma" variant from Iron Mt (Presence-Absence of genes).

**Additional file 2.**  Additional Information to Comparison of genomes of “E-plasma” Parys Mt to *C. divulgatum* and “E-plasma” variant from Iron Mt.

**Additional file 3. Supplementary Table S2.** Secreted proteins of "E-plasma".

**Additional file 4. Supplementary Table S3.** Pangenome analysis for the order *Thermoplasmatales*. Genome statistics.

**Additional file 5. Supplementary Table S4.** Comparison of "E-plasma" and *Cuniculiplasma* genomes against their CA and the LTCA.

**Additional file 6. Supplementary Table S5.** Comparison of *Ferroplasma/ Acidiplasma/ Picrophilus* "I-plasma genomes against their CA and the LTCA.

## Data Availability

Sequencing data (SRA archive) and biosample descriptions were deposited at DDBJ/ENA/GenBank under the Bioproject accession PRJNA474467. Specifically, the “E-plasma”-related MAG has been deposited at DDBJ/ENA/GenBank under the accession JARSDW000000000.
