# Supplementary material for: Evolutionary patterns of archaea predominant in acidic environment
Source: Environ Microbiome. 2023 Jul 18;18:61. doi: 10.1186/s40793-023-00518-5 (PMC10354927; doi:10.1186/s40793-023-00518-5)
Supplement: Supplementary file 2 — Additional file 2. Additional Information to Comparison of genomes of “E-plasma” Parys Mt to C. divulgatum and “E-plasma” variant from Iron Mt. [file 40793_2023_518_MOESM2_ESM.docx]

Supplementary Information

Evolutionary patterns of archaea predominant in acidic environment

Rafael Bargiela, Aleksei A. Korzhenkov, Owen A. McIntosh, Stepan V. Toshchakov, Michail M. Yakimov, Peter N. Golyshin and Olga V. Golyshina

Among “Information, Storage and Processing” category, some genes (Ribonuclease G and E, S-adenosylmethionine:tRNA ribosyltransferase-isomerase and an archaease, SHS2 domain) associated with Translation, Ribosomal structure and Biogenesis (J) were found in “E-plasma” genome, but not in *C. divulgatum* with other “E-plasma” genes from this subcategory represented mostly in higher copy numbers (Table S1).

“Replication, Recombination and Repair” subcategory (L)-associated genes were found to be in several copies in the “E-plasma” genome, specifically, archaeal DNA repair protein NreA and endonuclease IV), with endonuclease V detected only in “E-plasma” but not in both genomes of *C. divulgatum*.

Genes for another category, “Cellular Processes and Signalling”, particularly “Cell Wall/Membrane/Envelope biogenesis” (M) subcategory together with “Cell motility” (N) (genes connected with an archaellum assembly) and “Defence mechanisms” (V), were detected in higher numbers in “E-plasma” genomes. In more detail, the subcategory M genes for predicted pyridoxal phosphate-dependent enzyme apparently involved in regulation of cell wall biogenesis, a GDP-D-mannose dehydratase, a Phospholipase C, and an ADP-heptose:LPS heptosyltransferase family enzyme, with the latter protein homologous to bacterial counterparts, were overrepresented in the “E-plasma” genome in comparison to *C. divulgatum*.

Moreover, “Defence” subcategory genes identified in the “E-plasma” genome, but lacking in *C. divulgatum,* were predicted toxin of RNase A family, containing a vWA domain; a HicB family component of toxin-antitoxin system; an antitoxin, predicted nuclease of the RNAse H fold; a PemK/MazF family toxin; a CRISPR-Cas system-related protein Cas8, a large subunit of CASCADE complex and a HicB family component of toxin-antitoxin system. Furthermore, a CRISPR-associated protein Cas1, RHH/CopG family antitoxin and a cytotoxic translational repressor of toxin-antitoxin stability system were found in higher copy numbers in “E-plasma”.

Among “Metabolism” category genes, higher numbers were observed in the “E-plasma” in comparison to both *Cuniculiplasma* genomes for “Energy Production and Conversion” (C), “Nucleotide Transport and Metabolism” (F) and “Lipid Transport and Metabolism” (I) subcategories.

The subcategory C included a NADH:ubiquinone oxidoreductase subunit 4, a hydrogenase-4 membrane subunit HyfE, a Ni,Fe-hydrogenase III large subunit and a subunit G and a formate hydrogenlyase subunit 4 represented in multiple copies in the “E-plasma” genome. Additionally, among a C category, a gene encoding a flavodoxin reductase (a ferredoxin-NADPH reductase) family 1, lacking in both *Cuniculiplasma* genomes was noticed.

Subcategory F-affiliated genes in “E-plasma” included a nucleoside diphosphate kinase and a ribonucleotide reductase associated an ATP-cone domain shown in two copies and the presence of a deoxycytidine deaminase, absent in both *Cuniculiplasma* genomes.

Furthermore, the subcategory I revealed the presence of a dolichol kinase, a GtrA-like flippase, a mesaconyl-CoA hydratase (acyl dehydratase family) and a diacylglycerol kinase family enzyme, absent in both *Cuniculiplasma* genomes either. Additionally, we identified two sterol carrier protein gene copies in “E-plasma” with one protein being similar to *Cuniculiplasma* sterol carrier protein, and another to the predicted sterol carrier proteins from other *Thermoplasmatales*.
